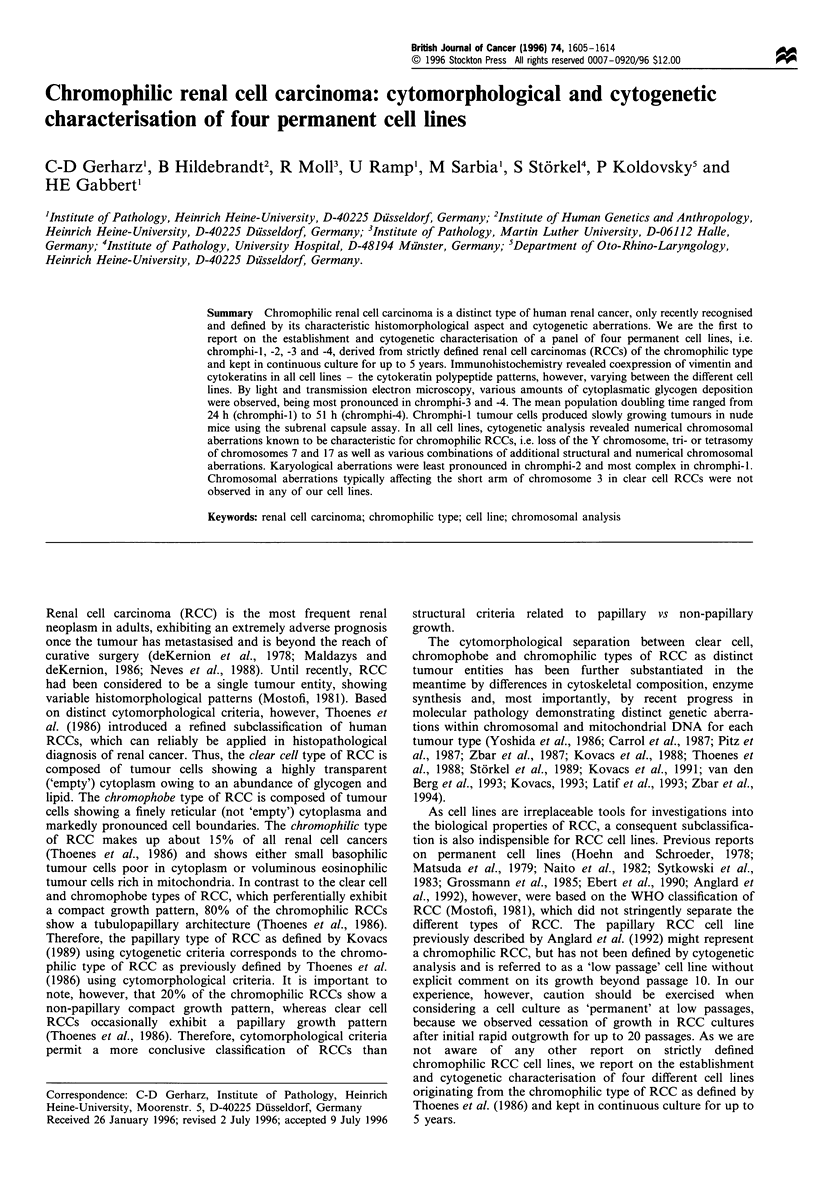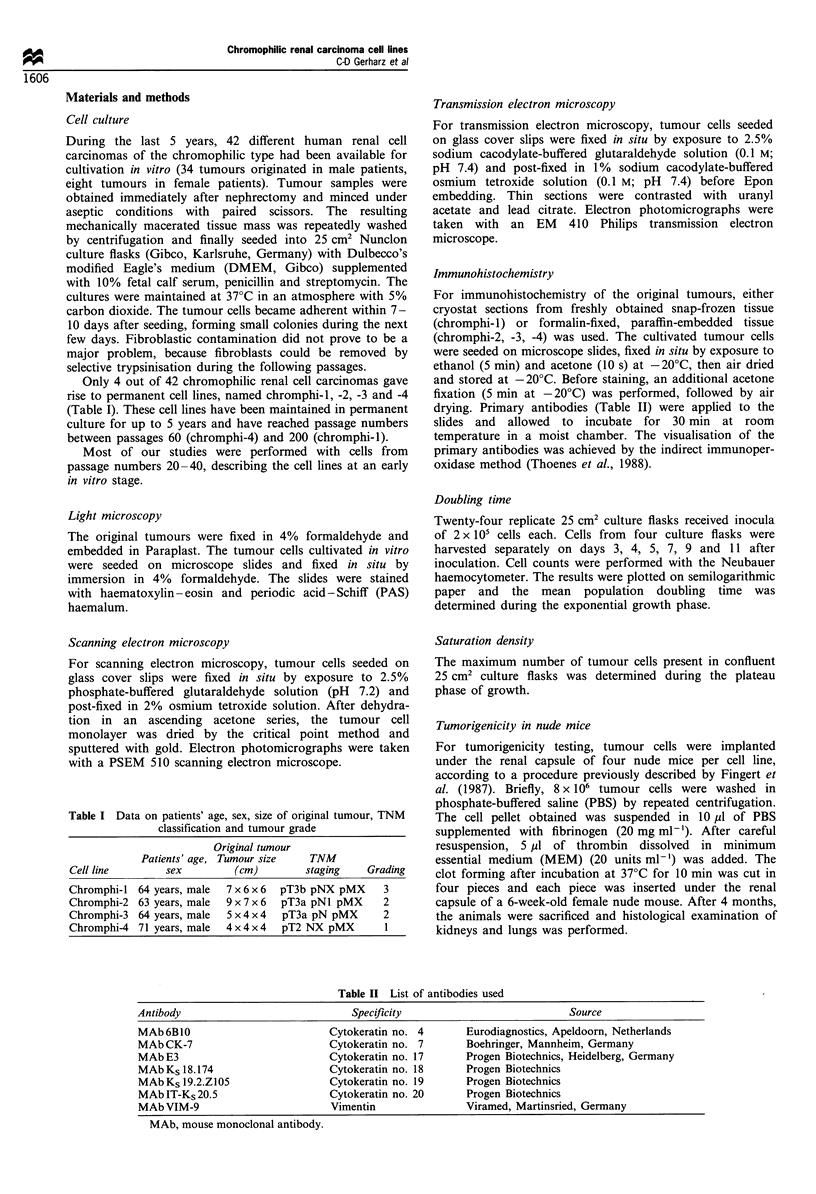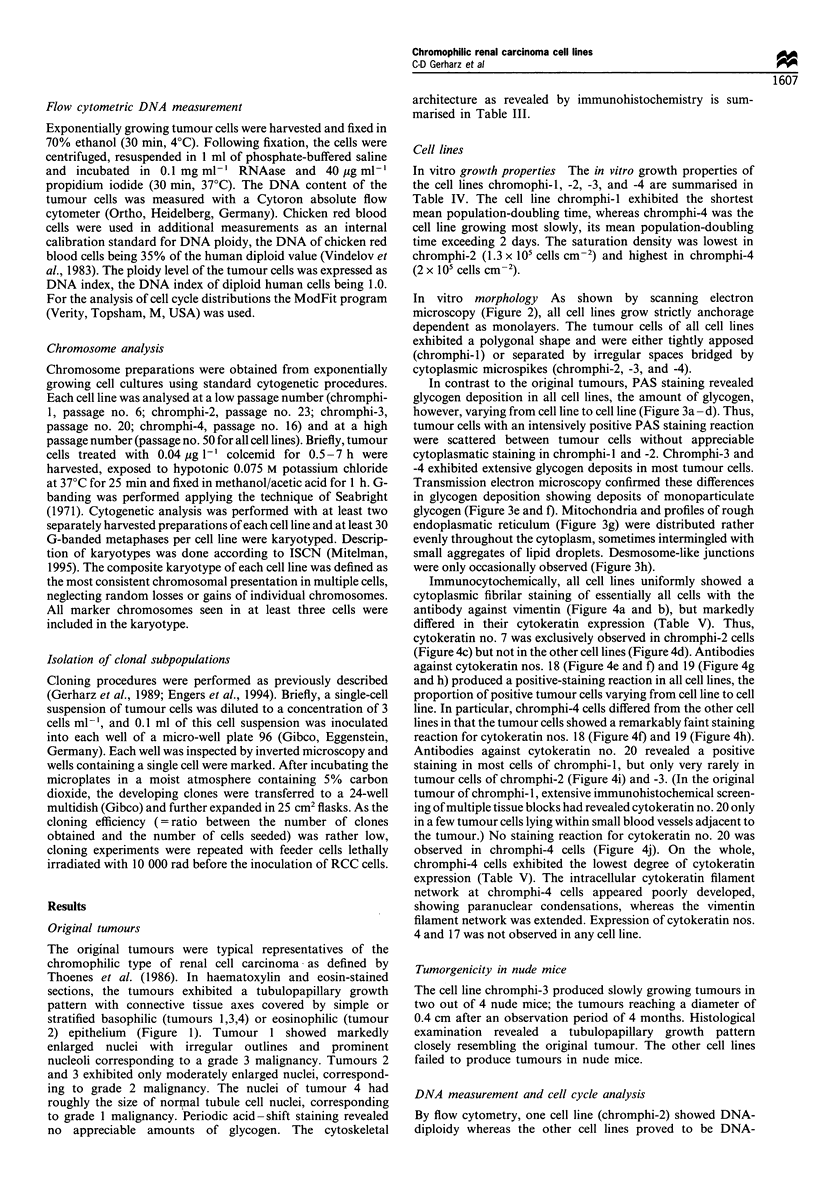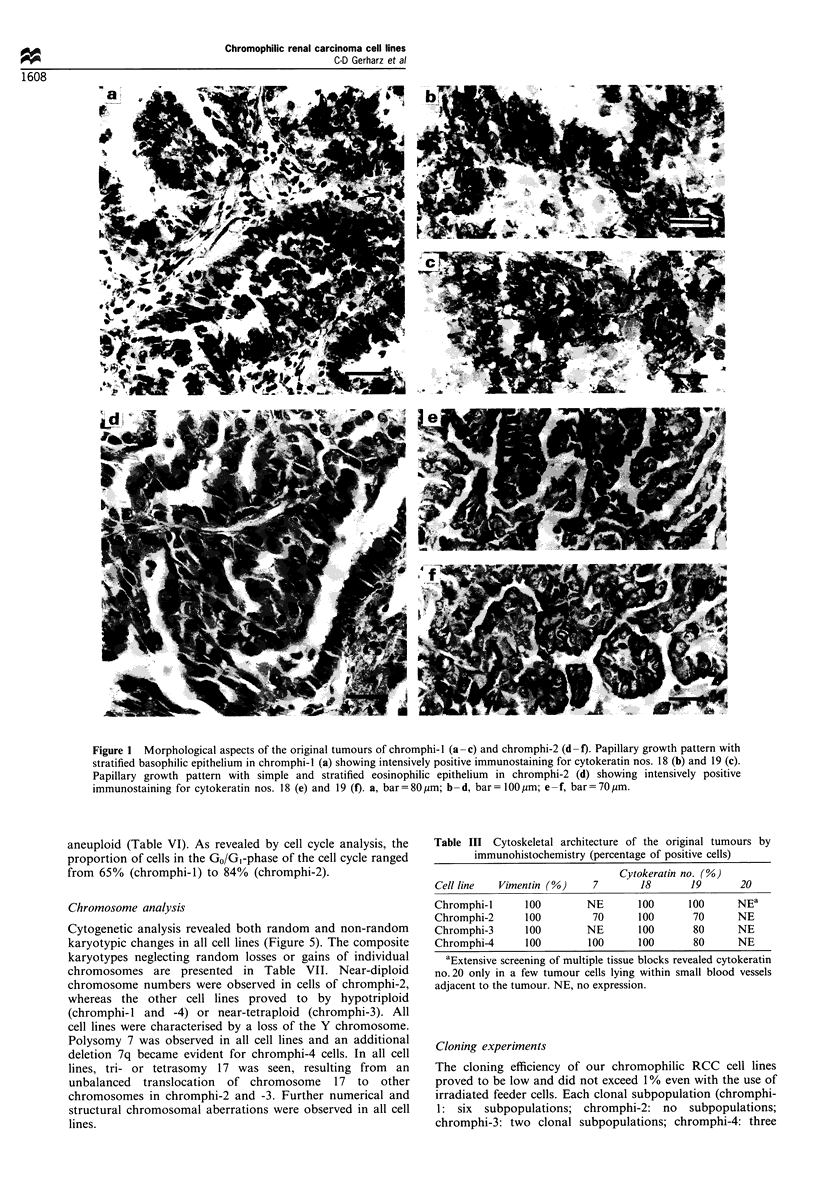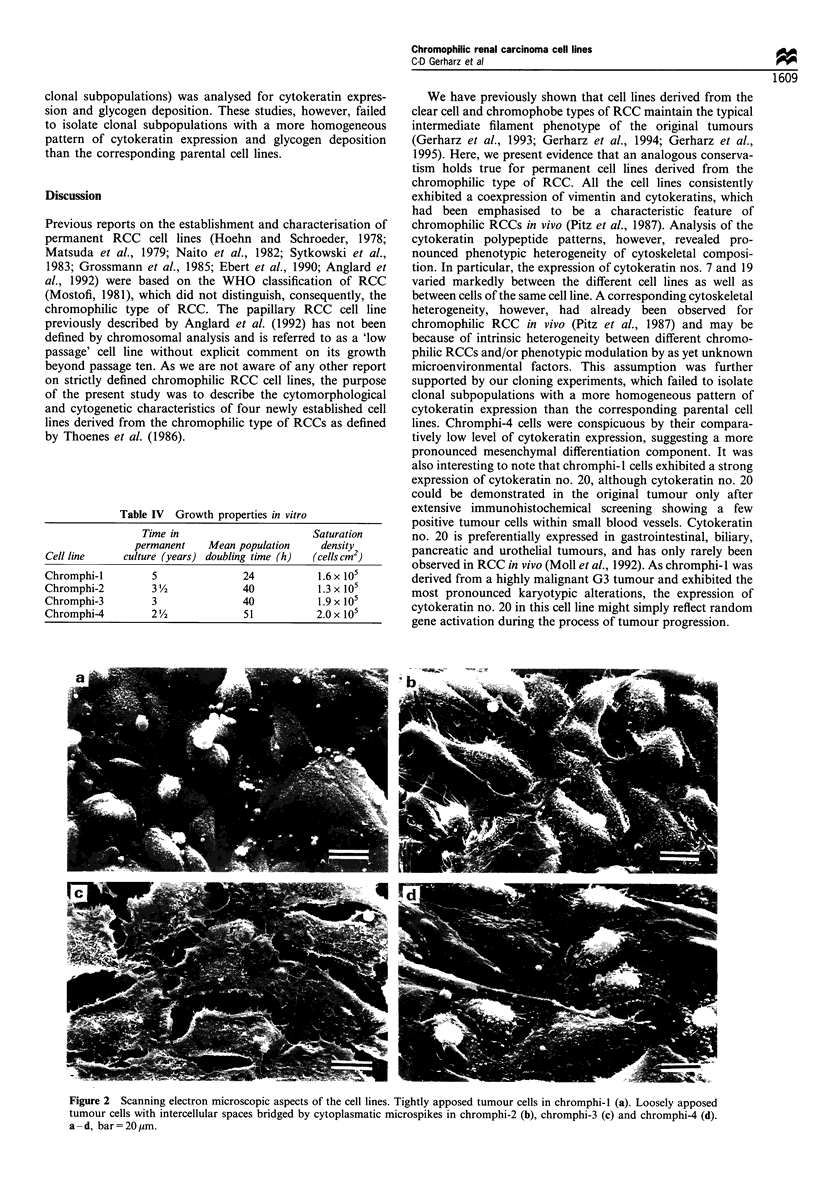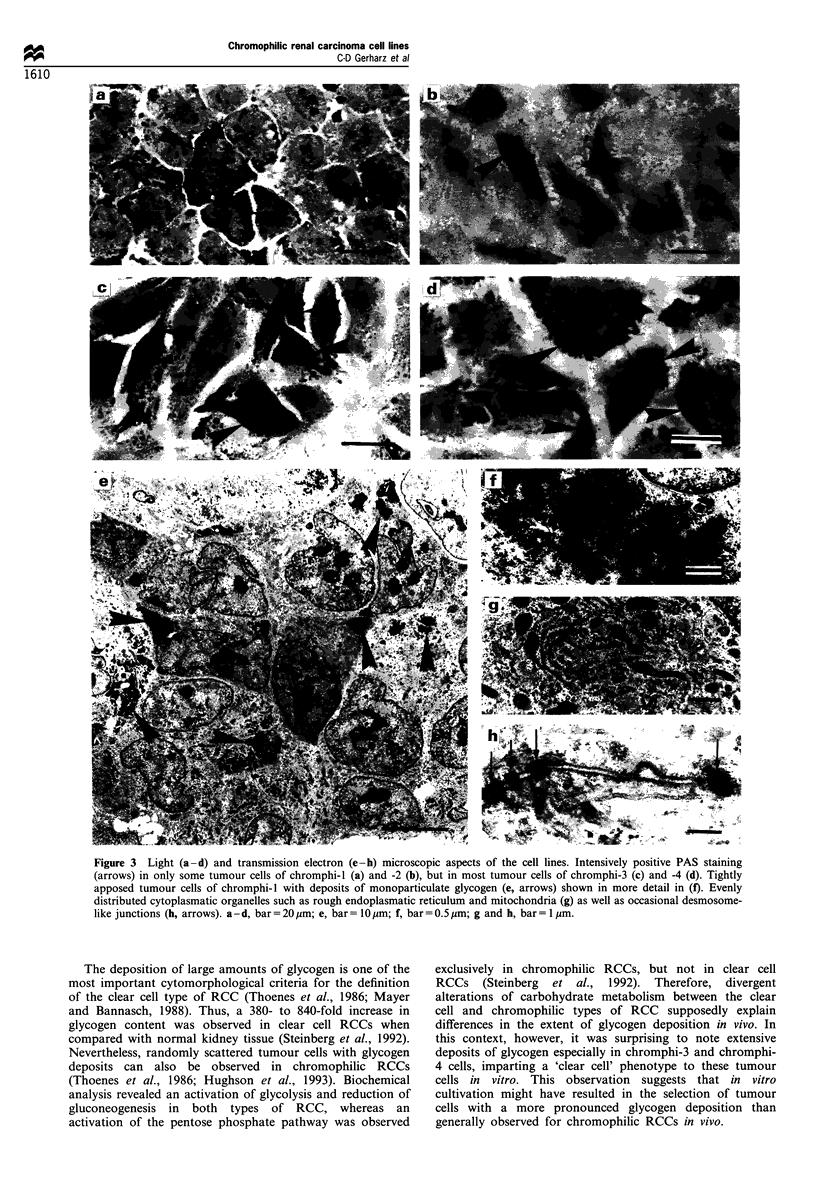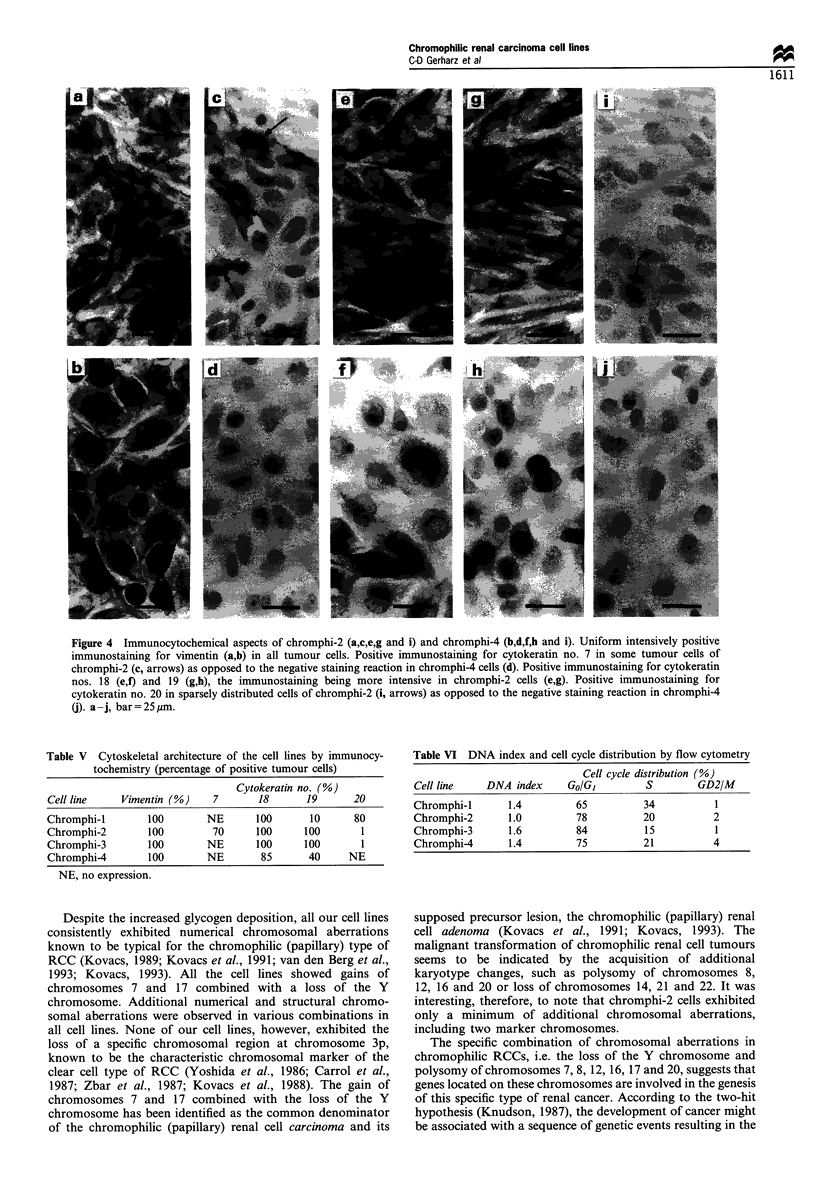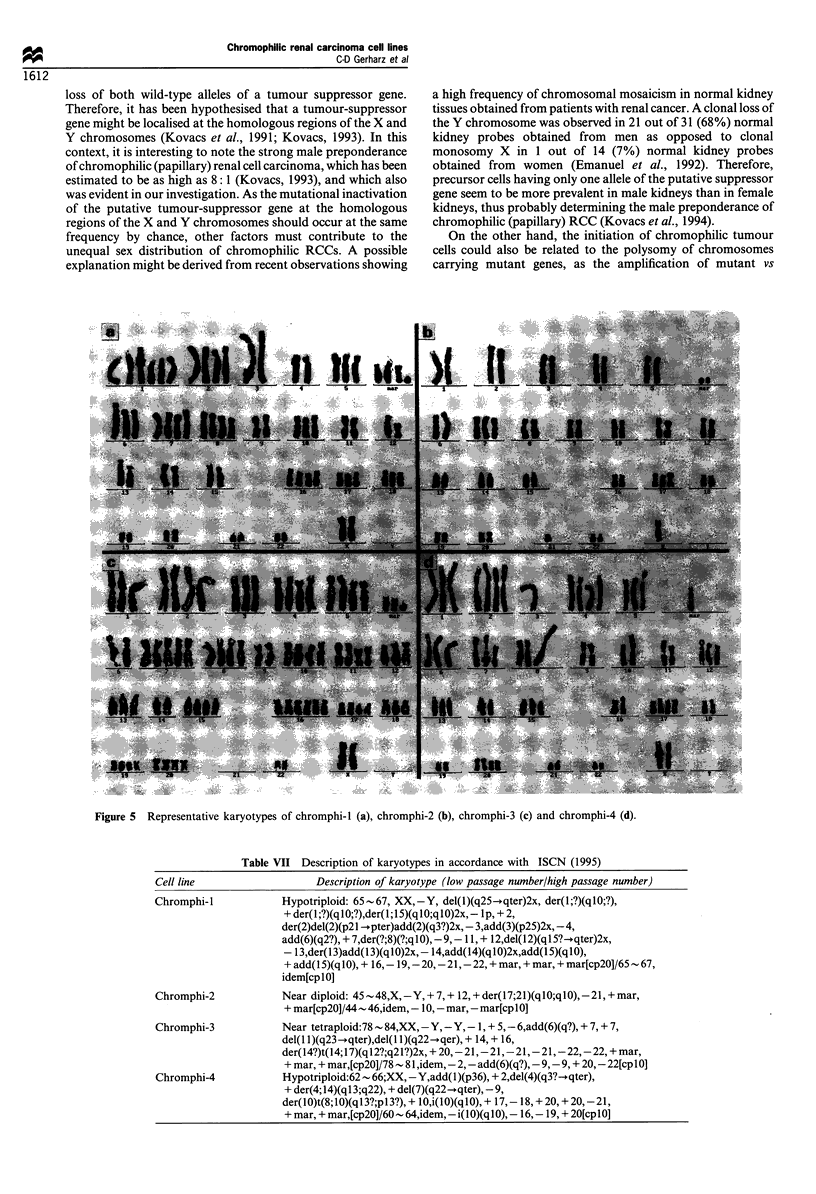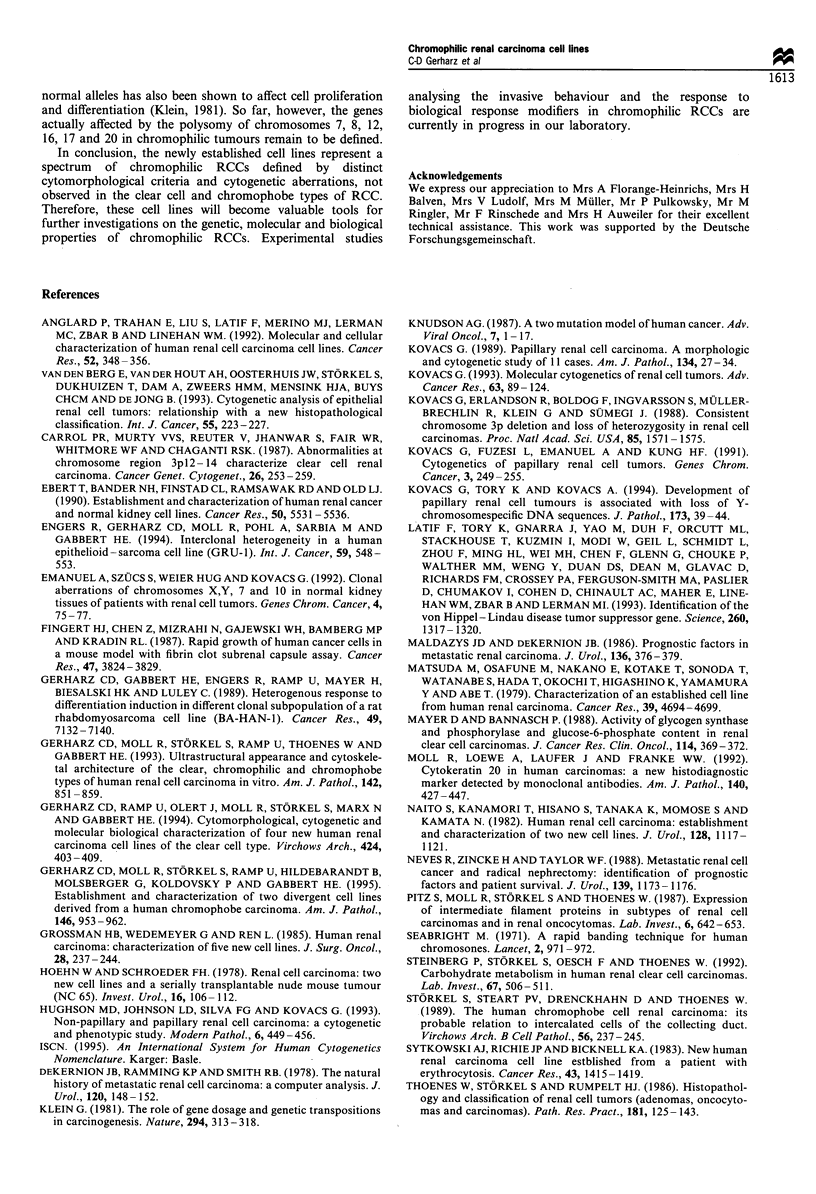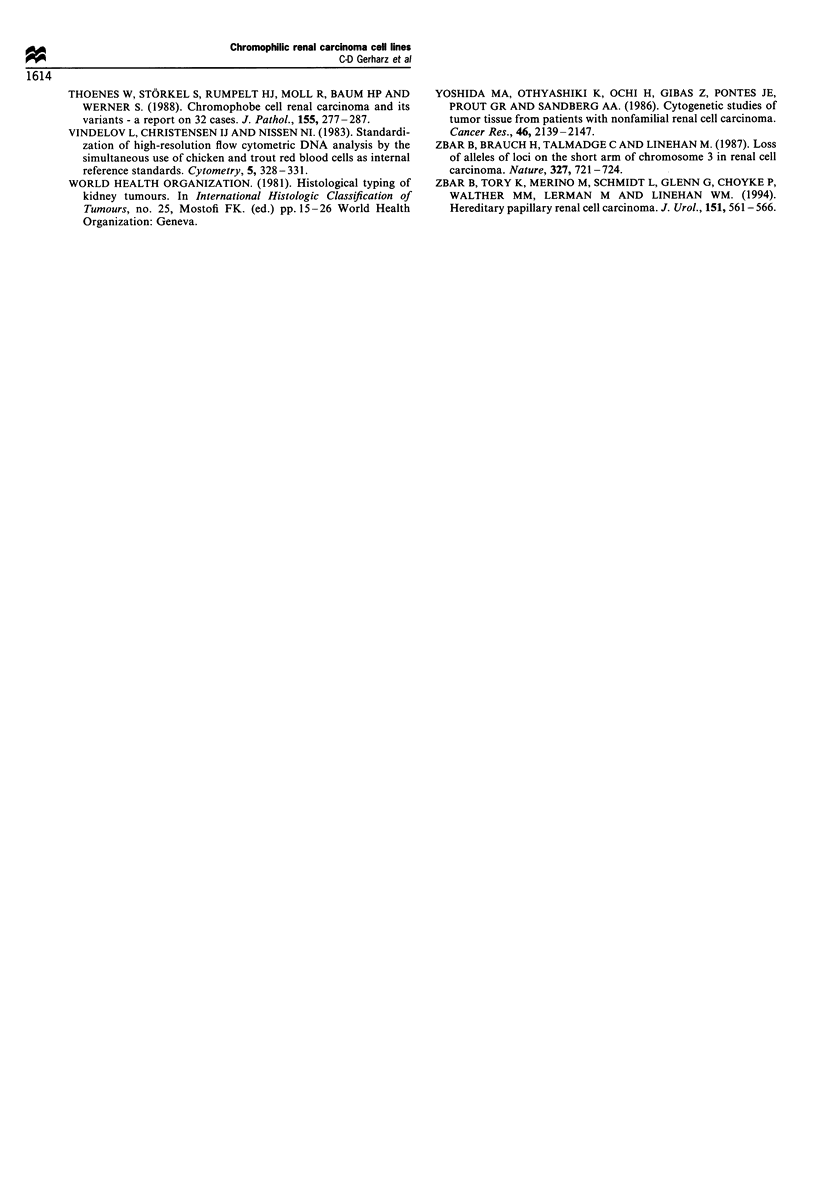# Chromophilic renal cell carcinoma: cytomorphological and cytogenetic characterisation of four permanent cell lines.

**DOI:** 10.1038/bjc.1996.596

**Published:** 1996-11

**Authors:** C. D. Gerharz, B. Hildebrandt, R. Moll, U. Ramp, M. Sarbia, S. Störkel, P. Koldovsky, H. E. Gabbert

**Affiliations:** Institute of Pathology, Heinrich Heine-University, Düsseldorf, Germany.

## Abstract

**Images:**


British Journal of Cancer (1996) 74, 1605-1614
© 1996 Stockton Press All rights reserved 0007-0920/96 $12.00

### Chromophilic renal cell carcinoma: cytomorphological and cytogenetic characterisation of four permanent cell lines

C-D Gerharz[1], B Hildebrandt[2], R Moll[3], U Ramp[1], M Sarbia[1], S Störkel[4], P Koldovsky[5] and HE Gabbert[1]

*[1]Institute of Pathology, Heinrich Heine-University, D-40225 Düsseldorf, Germany; [2]Institute of Human Genetics and Anthropology, Heinrich Heine-University, D-40225 Düsseldorf, Germany; [3]Institute of Pathology, Martin Luther University, D-06112 Halle, Germany; [4]Institute of Pathology, University Hospital, D-48194 Münster, Germany; [5]Department of Oto-Rhino-Laryngology, Heinrich Heine-University, D-40225 Düsseldorf, Germany.*

**Summary** Chromophilic renal cell carcinoma is a distinct type of human renal cancer, only recently recognised and defined by its characteristic histomorphological aspect and cytogenetic aberrations. We are the first to report on the establishment and cytogenetic characterisation of a panel of four permanent cell lines, i.e. chromphi-1, -2, -3 and -4, derived from strictly defined renal cell carcinomas (RCCs) of the chromophilic type and kept in continuous culture for up to 5 years. Immunohistochemistry revealed coexpression of vimentin and cytokeratins in all cell lines – the cytokeratin polypeptide patterns, however, varying between the different cell lines. By light and transmission electron microscopy, various amounts of cytoplasmatic glycogen deposition were observed, being most pronounced in chromphi-3 and -4. The mean population doubling time ranged from 24 h (chromphi-1) to 51 h (chromphi-4). Chromphi-1 tumour cells produced slowly growing tumours in nude mice using the subrenal capsule assay. In all cell lines, cytogenetic analysis revealed numerical chromosomal aberrations known to be characteristic for chromophilic RCCs, i.e. loss of the Y chromosome, tri- or tetrasomy of chromosomes 7 and 17 as well as various combinations of additional structural and numerical chromosomal aberrations. Karyological aberrations were least pronounced in chromphi-2 and most complex in chromphi-1. Chromosomal aberrations typically affecting the short arm of chromosome 3 in clear cell RCCs were not observed in any of our cell lines.

**Keywords:** renal cell carcinoma; chromophilic type; cell line; chromosomal analysis

Renal cell carcinoma (RCC) is the most frequent renal neoplasm in adults, exhibiting an extremely adverse prognosis once the tumour has metastasised and is beyond the reach of curative surgery (deKernion *et al.*, 1978; Maldazys and deKernion, 1986; Neves *et al.*, 1988). Until recently, RCC had been considered to be a single tumour entity, showing variable histomorphological patterns (Mostofi, 1981). Based on distinct cytomorphological criteria, however, Thoenes *et al.* (1986) introduced a refined subclassification of human RCCs, which can reliably be applied in histopathological diagnosis of renal cancer. Thus, the *clear cell* type of RCC is composed of tumour cells showing a highly transparent ('empty') cytoplasm owing to an abundance of glycogen and lipid. The *chromophobe* type of RCC is composed of tumour cells showing a finely reticular (not 'empty') cytoplasma and markedly pronounced cell boundaries. The *chromophilic* type of RCC makes up about 15% of all renal cell cancers (Thoenes *et al.*, 1986) and shows either small basophilic tumour cells poor in cytoplasm or voluminous eosinophilic tumour cells rich in mitochondria. In contrast to the clear cell and chromophobe types of RCC, which perferentially exhibit a compact growth pattern, 80% of the chromophilic RCCs show a tubulopapillary architecture (Thoenes *et al.*, 1986). Therefore, the papillary type of RCC as defined by Kovacs (1989) using cytogenetic criteria corresponds to the chromophilic type of RCC as previously defined by Thoenes *et al.* (1986) using cytomorphological criteria. It is important to note, however, that 20% of the chromophilic RCCs show a non-papillary compact growth pattern, whereas clear cell RCCs occasionally exhibit a papillary growth pattern (Thoenes *et al.*, 1986). Therefore, cytomorphological criteria permit a more conclusive classification of RCCs than structural criteria related to papillary *vs* non-papillary growth.

The cytomorphological separation between clear cell, chromophobe and chromophilic types of RCC as distinct tumour entities has been further substantiated in the meantime by differences in cytoskeletal composition, enzyme synthesis and, most importantly, by recent progress in molecular pathology demonstrating distinct genetic aberrations within chromosomal and mitochondrial DNA for each tumour type (Yoshida *et al.*, 1986; Carrol *et al.*, 1987; Pitz *et al.*, 1987; Zbar *et al.*, 1987; Kovacs *et al.*, 1988; Thoenes *et al.*, 1988; Störkel *et al.*, 1989; Kovacs *et al.*, 1991; van den Berg *et al.*, 1993; Kovacs, 1993; Latif *et al.*, 1993; Zbar *et al.*, 1994).

As cell lines are irreplaceable tools for investigations into the biological properties of RCC, a consequent subclassification is also indispensible for RCC cell lines. Previous reports on permanent cell lines (Hoehn and Schroeder, 1978; Matsuda *et al.*, 1979; Naito *et al.*, 1982; Sytkowski *et al.*, 1983; Grossmann *et al.*, 1985; Ebert *et al.*, 1990; Anglard *et al.*, 1992), however, were based on the WHO classification of RCC (Mostofi, 1981), which did not stringently separate the different types of RCC. The papillary RCC cell line previously described by Anglard *et al.* (1992) might represent a chromophilic RCC, but has not been defined by cytogenetic analysis and is referred to as a 'low passage' cell line without explicit comment on its growth beyond passage 10. In our experience, however, caution should be exercised when considering a cell culture as 'permanent' at low passages, because we observed cessation of growth in RCC cultures after initial rapid outgrowth for up to 20 passages. As we are not aware of any other report on strictly defined chromophilic RCC cell lines, we report on the establishment and cytogenetic characterisation of four different cell lines originating from the chromophilic type of RCC as defined by Thoenes *et al.* (1986) and kept in continuous culture for up to 5 years.

Correspondence: C-D Gerharz, Institute of Pathology, Heinrich Heine-University, Moorenstr. 5, D-40225 Düsseldorf, Germany
Received 26 January 1996; revised 2 July 1996; accepted 9 July 1996

### Materials and methods

### *Cell culture*

During the last 5 years, 42 different human renal cell carcinomas of the chromophilic type had been available for cultivation *in vitro* (34 tumours originated in male patients, eight tumours in female patients). Tumour samples were obtained immediately after nephrectomy and minced under aseptic conditions with paired scissors. The resulting mechanically macerated tissue mass was repeatedly washed by centrifugation and finally seeded into 25 $cm^2$ Nunclon culture flasks (Gibco, Karlsruhe, Germany) with Dulbecco's modified Eagle's medium (DMEM, Gibco) supplemented with 10% fetal calf serum, penicillin and streptomycin. The cultures were maintained at 37°C in an atmosphere with 5% carbon dioxide. The tumour cells became adherent within 7–10 days after seeding, forming small colonies during the next few days. Fibroblastic contamination did not prove to be a major problem, because fibroblasts could be removed by selective trypsinisation during the following passages.

Only 4 out of 42 chromophilic renal cell carcinomas gave rise to permanent cell lines, named chromphi-1, -2, -3 and -4 (Table I). These cell lines have been maintained in permanent culture for up to 5 years and have reached passage numbers between passages 60 (chromphi-4) and 200 (chromphi-1).

Most of our studies were performed with cells from passage numbers 20–40, describing the cell lines at an early *in vitro* stage.

### *Light microscopy*

The original tumours were fixed in 4% formaldehyde and embedded in Paraplast. The tumour cells cultivated *in vitro* were seeded on microscope slides and fixed *in situ* by immersion in 4% formaldehyde. The slides were stained with haematoxylin–eosin and periodic acid–Schiff (PAS) haemalum.

### *Scanning electron microscopy*

For scanning electron microscopy, tumour cells seeded on glass cover slips were fixed *in situ* by exposure to 2.5% phosphate-buffered glutaraldehyde solution (pH 7.2) and post-fixed in 2% osmium tetroxide solution. After dehydration in an ascending acetone series, the tumour cell monolayer was dried by the critical point method and sputtered with gold. Electron photomicrographs were taken with a PSEM 510 scanning electron microscope.

**Table I** Data on patients' age, sex, size of original tumour, TNM classification and tumour grade

| *Cell line* | *Patients' age, sex* | *Original tumour Tumour size (cm)* | *TNM staging* | *Grading* |
|---|---|---|---|---|
| Chromphi-1 | 64 years, male | 7×6×6 | pT3b pNX pMX | 3 |
| Chromphi-2 | 63 years, male | 9×7×6 | pT3a pN1 pMX | 2 |
| Chromphi-3 | 64 years, male | 5×4×4 | pT3a pN pMX | 2 |
| Chromphi-4 | 71 years, male | 4×4×4 | pT2 NX pMX | 1 |

### *Transmission electron microscopy*

For transmission electron microscopy, tumour cells seeded on glass cover slips were fixed *in situ* by exposure to 2.5% sodium cacodylate-buffered glutaraldehyde solution (0.1 M; pH 7.4) and post-fixed in 1% sodium cacodylate-buffered osmium tetroxide solution (0.1 M; pH 7.4) before Epon embedding. Thin sections were contrasted with uranyl acetate and lead citrate. Electron photomicrographs were taken with an EM 410 Philips transmission electron microscope.

### *Immunohistochemistry*

For immunohistochemistry of the original tumours, either cryostat sections from freshly obtained snap-frozen tissue (chromphi-1) or formalin-fixed, paraffin-embedded tissue (chromphi-2, -3, -4) was used. The cultivated tumour cells were seeded on microscope slides, fixed *in situ* by exposure to ethanol (5 min) and acetone (10 s) at −20°C, then air dried and stored at −20°C. Before staining, an additional acetone fixation (5 min at −20°C) was performed, followed by air drying. Primary antibodies (Table II) were applied to the slides and allowed to incubate for 30 min at room temperature in a moist chamber. The visualisation of the primary antibodies was achieved by the indirect immunoperoxidase method (Thoenes *et al.*, 1988).

### *Doubling time*

Twenty-four replicate 25 $cm^2$ culture flasks received inocula of $2 \times 10^5$ cells each. Cells from four culture flasks were harvested separately on days 3, 4, 5, 7, 9 and 11 after inoculation. Cell counts were performed with the Neubauer haemocytometer. The results were plotted on semilogarithmic paper and the mean population doubling time was determined during the exponential growth phase.

### *Saturation density*

The maximum number of tumour cells present in confluent 25 $cm^2$ culture flasks was determined during the plateau phase of growth.

### *Tumorigenicity in nude mice*

For tumorigenicity testing, tumour cells were implanted under the renal capsule of four nude mice per cell line, according to a procedure previously described by Fingert *et al.* (1987). Briefly, $8 \times 10^6$ tumour cells were washed in phosphate-buffered saline (PBS) by repeated centrifugation. The cell pellet obtained was suspended in 10 $\mu l$ of PBS supplemented with fibrinogen (20 mg $ml^{-1}$). After careful resuspension, 5 $\mu l$ of thrombin dissolved in minimum essential medium (MEM) (20 units $ml^{-1}$) was added. The clot forming after incubation at 37°C for 10 min was cut in four pieces and each piece was inserted under the renal capsule of a 6-week-old female nude mouse. After 4 months, the animals were sacrificed and histological examination of kidneys and lungs was performed.

**Table II** List of antibodies used

| *Antibody* | *Specificity* | *Source* |
|---|---|---|
| MAb 6B10 | Cytokeratin no. 4 | Eurodiagnostics, Apeldoorn, Netherlands |
| MAb CK-7 | Cytokeratin no. 7 | Boehringer, Mannheim, Germany |
| MAb E3 | Cytokeratin no. 17 | Progen Biotechnics, Heidelberg, Germany |
| MAb $K_S$ 18.174 | Cytokeratin no. 18 | Progen Biotechnics |
| MAb $K_S$ 19.2.Z105 | Cytokeratin no. 19 | Progen Biotechnics |
| MAb IT-$K_S$ 20.5 | Cytokeratin no. 20 | Progen Biotechnics |
| MAb VIM-9 | Vimentin | Viramed, Martinsried, Germany |

MAb, mouse monoclonal antibody.

*Flow cytometric DNA measurement*

Exponentially growing tumour cells were harvested and fixed in 70% ethanol (30 min, 4°C). Following fixation, the cells were centrifuged, resuspended in 1 ml of phosphate-buffered saline and incubated in 0.1 mg $ml^{-1}$ RNAase and 40 $\mu g$ $ml^{-1}$ propidium iodide (30 min, 37°C). The DNA content of the tumour cells was measured with a Cytoron absolute flow cytometer (Ortho, Heidelberg, Germany). Chicken red blood cells were used in additional measurements as an internal calibration standard for DNA ploidy, the DNA of chicken red blood cells being 35% of the human diploid value (Vindelov *et al.*, 1983). The ploidy level of the tumour cells was expressed as DNA index, the DNA index of diploid human cells being 1.0. For the analysis of cell cycle distributions the ModFit program (Verity, Topsham, M, USA) was used.

*Chromosome analysis*

Chromosome preparations were obtained from exponentially growing cell cultures using standard cytogenetic procedures. Each cell line was analysed at a low passage number (chromphi-1, passage no. 6; chromphi-2, passage no. 23; chromphi-3, passage no. 20; chromphi-4, passage no. 16) and at a high passage number (passage no. 50 for all cell lines). Briefly, tumour cells treated with 0.04 $\mu g$ $l^{-1}$ colcemid for 0.5–7 h were harvested, exposed to hypotonic 0.075 M potassium chloride at 37°C for 25 min and fixed in methanol/acetic acid for 1 h. G-banding was performed applying the technique of Seabright (1971). Cytogenetic analysis was performed with at least two separately harvested preparations of each cell line and at least 30 G-banded metaphases per cell line were karyotyped. Description of karyotypes was done according to ISCN (Mitelman, 1995). The composite karyotype of each cell line was defined as the most consistent chromosomal presentation in multiple cells, neglecting random losses or gains of individual chromosomes. All marker chromosomes seen in at least three cells were included in the karyotype.

*Isolation of clonal subpopulations*

Cloning procedures were performed as previously described (Gerharz *et al.*, 1989; Engers *et al.*, 1994). Briefly, a single-cell suspension of tumour cells was diluted to a concentration of 3 cells $ml^{-1}$, and 0.1 ml of this cell suspension was inoculated into each well of a micro-well plate 96 (Gibco, Eggenstein, Germany). Each well was inspected by inverted microscopy and wells containing a single cell were marked. After incubating the microplates in a moist atmosphere containing 5% carbon dioxide, the developing clones were transferred to a 24-well multidish (Gibco) and further expanded in 25 $cm^2$ flasks. As the cloning efficiency (=ratio between the number of clones obtained and the number of cells seeded) was rather low, cloning experiments were repeated with feeder cells lethally irradiated with 10 000 rad before the inoculation of RCC cells.

### Results

*Original tumours*

The original tumours were typical representatives of the chromophilic type of renal cell carcinoma as defined by Thoenes *et al.* (1986). In haematoxylin and eosin-stained sections, the tumours exhibited a tubulopapillary growth pattern with connective tissue axes covered by simple or stratified basophilic (tumours 1,3,4) or eosinophilic (tumour 2) epithelium (Figure 1). Tumour 1 showed markedly enlarged nuclei with irregular outlines and prominent nucleoli corresponding to a grade 3 malignancy. Tumours 2 and 3 exhibited only moderately enlarged nuclei, corresponding to grade 2 malignancy. The nuclei of tumour 4 had roughly the size of normal tubule cell nuclei, corresponding to grade 1 malignancy. Periodic acid–shift staining revealed no appreciable amounts of glycogen. The cytoskeletal architecture as revealed by immunohistochemistry is summarised in Table III.

*Cell lines*

In vitro *growth properties* The *in vitro* growth properties of the cell lines chromophi-1, -2, -3, and -4 are summarised in Table IV. The cell line chromphi-1 exhibited the shortest mean population-doubling time, whereas chromphi-4 was the cell line growing most slowly, its mean population-doubling time exceeding 2 days. The saturation density was lowest in chromphi-2 ($1.3 \times 10^5$ cells $cm^{-2}$) and highest in chromphi-4 ($2 \times 10^5$ cells $cm^{-2}$).

In vitro *morphology* As shown by scanning electron microscopy (Figure 2), all cell lines grow strictly anchorage dependent as monolayers. The tumour cells of all cell lines exhibited a polygonal shape and were either tightly apposed (chromphi-1) or separated by irregular spaces bridged by cytoplasmic microspikes (chromphi-2, -3, and -4).

In contrast to the original tumours, PAS staining revealed glycogen deposition in all cell lines, the amount of glycogen, however, varying from cell line to cell line (Figure 3a–d). Thus, tumour cells with an intensively positive PAS staining reaction were scattered between tumour cells without appreciable cytoplasmatic staining in chromphi-1 and -2. Chromphi-3 and -4 exhibited extensive glycogen deposits in most tumour cells. Transmission electron microscopy confirmed these differences in glycogen deposition showing deposits of monoparticulate glycogen (Figure 3e and f). Mitochondria and profiles of rough endoplasmatic reticulum (Figure 3g) were distributed rather evenly throughout the cytoplasm, sometimes intermingled with small aggregates of lipid droplets. Desmosome-like junctions were only occasionally observed (Figure 3h).

Immunocytochemically, all cell lines uniformly showed a cytoplasmic fibrilar staining of essentially all cells with the antibody against vimentin (Figure 4a and b), but markedly differed in their cytokeratin expression (Table V). Thus, cytokeratin no. 7 was exclusively observed in chromphi-2 cells (Figure 4c) but not in the other cell lines (Figure 4d). Antibodies against cytokeratin nos. 18 (Figure 4e and f) and 19 (Figure 4g and h) produced a positive-staining reaction in all cell lines, the proportion of positive tumour cells varying from cell line to cell line. In particular, chromphi-4 cells differed from the other cell lines in that the tumour cells showed a remarkably faint staining reaction for cytokeratin nos. 18 (Figure 4f) and 19 (Figure 4h). Antibodies against cytokeratin no. 20 revealed a positive staining in most cells of chromphi-1, but only very rarely in tumour cells of chromphi-2 (Figure 4i) and -3. (In the original tumour of chromphi-1, extensive immunohistochemical screening of multiple tissue blocks had revealed cytokeratin no. 20 only in a few tumour cells lying within small blood vessels adjacent to the tumour.) No staining reaction for cytokeratin no. 20 was observed in chromphi-4 cells (Figure 4j). On the whole, chromphi-4 cells exhibited the lowest degree of cytokeratin expression (Table V). The intracellular cytokeratin filament network at chromphi-4 cells appeared poorly developed, showing paranuclear condensations, whereas the vimentin filament network was extended. Expression of cytokeratin nos. 4 and 17 was not observed in any cell line.

*Tumorgenicity in nude mice*

The cell line chromphi-3 produced slowly growing tumours in two out of 4 nude mice; the tumours reaching a diameter of 0.4 cm after an observation period of 4 months. Histological examination revealed a tubulopapillary growth pattern closely resembling the original tumour. The other cell lines failed to produce tumours in nude mice.

*DNA measurement and cell cycle analysis*

By flow cytometry, one cell line (chromphi-2) showed DNA-diploidy whereas the other cell lines proved to be DNA-

**Figure 1** Morphological aspects of the original tumours of chromphi-1 (**a**–**c**) and chromphi-2 (**d**–**f**). Papillary growth pattern with stratified basophilic epithelium in chromphi-1 (**a**) showing intensively positive immunostaining for cytokeratin nos. 18 (**b**) and 19 (**c**). Papillary growth pattern with simple and stratified eosinophilic epithelium in chromphi-2 (**d**) showing intensively positive immunostaining for cytokeratin nos. 18 (**e**) and 19 (**f**). **a**, bar = 80 μm; **b**–**d**, bar = 100 μm; **e**–**f**, bar = 70 μm.

aneuploid (Table VI). As revealed by cell cycle analysis, the proportion of cells in the $G_0/G_1$-phase of the cell cycle ranged from 65% (chromphi-1) to 84% (chromphi-2).

*Chromosome analysis*

Cytogenetic analysis revealed both random and non-random karyotypic changes in all cell lines (Figure 5). The composite karyotypes neglecting random losses or gains of individual chromosomes are presented in Table VII. Near-diploid chromosome numbers were observed in cells of chromphi-2, whereas the other cell lines proved to by hypotriploid (chromphi-1 and -4) or near-tetraploid (chromphi-3). All cell lines were characterised by a loss of the Y chromosome. Polysomy 7 was observed in all cell lines and an additional deletion 7q became evident for chromphi-4 cells. In all cell lines, tri- or tetrasomy 17 was seen, resulting from an unbalanced translocation of chromosome 17 to other chromosomes in chromphi-2 and -3. Further numerical and structural chromosomal aberrations were observed in all cell lines.

**Table III** Cytoskeletal architecture of the original tumours by immunohistochemistry (percentage of positive cells)

| | | Cytokeratin no. (%) | | | |
|---|---|---|---|---|---|
| *Cell line* | *Vimentin (%)* | *7* | *18* | *19* | *20* |
| Chromphi-1 | 100 | NE | 100 | 100 | NE[a] |
| Chromphi-2 | 100 | 70 | 100 | 70 | NE |
| Chromphi-3 | 100 | NE | 100 | 80 | NE |
| Chromphi-4 | 100 | 100 | 100 | 80 | NE |

[a]Extensive screening of multiple tissue blocks revealed cytokeratin no. 20 only in a few tumour cells lying within small blood vessels adjacent to the tumour. NE, no expression.

*Cloning experiments*

The cloning efficiency of our chromophilic RCC cell lines proved to be low and did not exceed 1% even with the use of irradiated feeder cells. Each clonal subpopulation (chromphi-1: six subpopulations; chromphi-2: no subpopulations; chromphi-3: two clonal subpopulations; chromphi-4: three clonal subpopulations) was analysed for cytokeratin expression and glycogen deposition. These studies, however, failed to isolate clonal subpopulations with a more homogeneous pattern of cytokeratin expression and glycogen deposition than the corresponding parental cell lines.

### Discussion

Previous reports on the establishment and characterisation of permanent RCC cell lines (Hoehn and Schroeder, 1978; Matsuda *et al.*, 1979; Naito *et al.*, 1982; Sytkowski *et al.*, 1983; Grossmann *et al.*, 1985; Ebert *et al.*, 1990; Anglard *et al.*, 1992) were based on the WHO classification of RCC (Mostofi, 1981), which did not distinguish, consequently, the chromophilic type of RCC. The papillary RCC cell line previously described by Anglard *et al.* (1992) has not been defined by chromosomal analysis and is referred to as a 'low passage' cell line without explicit comment on its growth beyond passage ten. As we are not aware of any other report on strictly defined chromophilic RCC cell lines, the purpose of the present study was to describe the cytomorphological and cytogenetic characteristics of four newly established cell lines derived from the chromophilic type of RCCs as defined by Thoenes *et al.* (1986).

**Table IV** Growth properties *in vitro*

| *Cell line* | *Time in permanent culture (years)* | *Mean population doubling time (h)* | *Saturation density (cells cm$^2$)* |
|---|---|---|---|
| Chromphi-1 | 5 | 24 | $1.6 \times 10^5$ |
| Chromphi-2 | 3½ | 40 | $1.3 \times 10^5$ |
| Chromphi-3 | 3 | 40 | $1.9 \times 10^5$ |
| Chromphi-4 | 2½ | 51 | $2.0 \times 10^5$ |

We have previously shown that cell lines derived from the clear cell and chromophobe types of RCC maintain the typical intermediate filament phenotype of the original tumours (Gerharz *et al.*, 1993; Gerharz *et al.*, 1994; Gerharz *et al.*, 1995). Here, we present evidence that an analogous conservatism holds true for permanent cell lines derived from the chromophilic type of RCC. All the cell lines consistently exhibited a coexpression of vimentin and cytokeratins, which had been emphasised to be a characteristic feature of chromophilic RCCs *in vivo* (Pitz *et al.*, 1987). Analysis of the cytokeratin polypeptide patterns, however, revealed pronounced phenotypic heterogeneity of cytoskeletal composition. In particular, the expression of cytokeratin nos. 7 and 19 varied markedly between the different cell lines as well as between cells of the same cell line. A corresponding cytoskeletal heterogeneity, however, had already been observed for chromophilic RCC *in vivo* (Pitz *et al.*, 1987) and may be because of intrinsic heterogeneity between different chromophilic RCCs and/or phenotypic modulation by as yet unknown microenvironmental factors. This assumption was further supported by our cloning experiments, which failed to isolate clonal subpopulations with a more homogeneous pattern of cytokeratin expression than the corresponding parental cell lines. Chromphi-4 cells were conspicuous by their comparatively low level of cytokeratin expression, suggesting a more pronounced mesenchymal differentiation component. It was also interesting to note that chromphi-1 cells exhibited a strong expression of cytokeratin no. 20, although cytokeratin no. 20 could be demonstrated in the original tumour only after extensive immunohistochemical screening showing a few positive tumour cells within small blood vessels. Cytokeratin no. 20 is preferentially expressed in gastrointestinal, biliary, pancreatic and urothelial tumours, and has only rarely been observed in RCC *in vivo* (Moll *et al.*, 1992). As chromphi-1 was derived from a highly malignant G3 tumour and exhibited the most pronounced karyotypic alterations, the expression of cytokeratin no. 20 in this cell line might simply reflect random gene activation during the process of tumour progression.

**Figure 2** Scanning electron microscopic aspects of the cell lines. Tightly apposed tumour cells in chromphi-1 (**a**). Loosely apposed tumour cells with intercellular spaces bridged by cytoplasmatic microspikes in chromphi-2 (**b**), chromphi-3 (**c**) and chromphi-4 (**d**). **a**–**d**, bar = 20 $\mu$m.

**Figure 3** Light (**a**–**d**) and transmission electron (**e**–**h**) microscopic aspects of the cell lines. Intensively positive PAS staining (arrows) in only some tumour cells of chromphi-1 (**a**) and -2 (**b**), but in most tumour cells of chromphi-3 (**c**) and -4 (**d**). Tightly apposed tumour cells of chromphi-1 with deposits of monoparticulate glycogen (**e**, arrows) shown in more detail in (**f**). Evenly distributed cytoplasmatic organelles such as rough endoplasmatic reticulum and mitochondria (**g**) as well as occasional desmosome-like junctions (**h**, arrows). **a**–**d**, bar = 20 $\mu$m; **e**, bar = 10 $\mu$m; **f**, bar = 0.5 $\mu$m; **g** and **h**, bar = 1 $\mu$m.

The deposition of large amounts of glycogen is one of the most important cytomorphological criteria for the definition of the clear cell type of RCC (Thoenes *et al.*, 1986; Mayer and Bannasch, 1988). Thus, a 380- to 840-fold increase in glycogen content was observed in clear cell RCCs when compared with normal kidney tissue (Steinberg *et al.*, 1992). Nevertheless, randomly scattered tumour cells with glycogen deposits can also be observed in chromophilic RCCs (Thoenes *et al.*, 1986; Hughson *et al.*, 1993). Biochemical analysis revealed an activation of glycolysis and reduction of gluconeogenesis in both types of RCC, whereas an activation of the pentose phosphate pathway was observed exclusively in chromophilic RCCs, but not in clear cell RCCs (Steinberg *et al.*, 1992). Therefore, divergent alterations of carbohydrate metabolism between the clear cell and chromophilic types of RCC supposedly explain differences in the extent of glycogen deposition *in vivo*. In this context, however, it was surprising to note extensive deposits of glycogen especially in chromphi-3 and chromphi-4 cells, imparting a 'clear cell' phenotype to these tumour cells *in vitro*. This observation suggests that *in vitro* cultivation might have resulted in the selection of tumour cells with a more pronounced glycogen deposition than generally observed for chromophilic RCCs *in vivo*.

**Figure 4** Immunocytochemical aspects of chromphi-2 (**a,c,e,g** and **i**) and chromphi-4 (**b,d,f,h** and **i**). Uniform intensively positive immunostaining for vimentin (**a,b**) in all tumour cells. Positive immunostaining for cytokeratin no. 7 in some tumour cells of chromphi-2 (**c**, arrows) as opposed to the negative staining reaction in chromphi-4 cells (**d**). Positive immunostaining for cytokeratin nos. 18 (**e,f**) and 19 (**g,h**), the immunostaining being more intensive in chromphi-2 cells (**e,g**). Positive immunostaining for cytokeratin no. 20 in sparsely distributed cells of chromphi-2 (**i**, arrows) as opposed to the negative staining reaction in chromphi-4 (**j**). **a**–**j**, bar = 25 μm.

**Table V** Cytoskeletal architecture of the cell lines by immunocytochemistry (percentage of positive tumour cells)

| *Cell line* | *Vimentin (%)* | *Cytokeratin no. (%)* 7 | 18 | 19 | 20 |
|---|---|---|---|---|---|
| Chromphi-1 | 100 | NE | 100 | 10 | 80 |
| Chromphi-2 | 100 | 70 | 100 | 100 | 1 |
| Chromphi-3 | 100 | NE | 100 | 100 | 1 |
| Chromphi-4 | 100 | NE | 85 | 40 | NE |

NE, no expression.

**Table VI** DNA index and cell cycle distribution by flow cytometry

| *Cell line* | *DNA index* | *Cell cycle distribution (%)* $G_0/G_1$ | *S* | *GD2/M* |
|---|---|---|---|---|
| Chromphi-1 | 1.4 | 65 | 34 | 1 |
| Chromphi-2 | 1.0 | 78 | 20 | 2 |
| Chromphi-3 | 1.6 | 84 | 15 | 1 |
| Chromphi-4 | 1.4 | 75 | 21 | 4 |

Despite the increased glycogen deposition, all our cell lines consistently exhibited numerical chromosomal aberrations known to be typical for the chromophilic (papillary) type of RCC (Kovacs, 1989; Kovacs *et al.*, 1991; van den Berg *et al.*, 1993; Kovacs, 1993). All the cell lines showed gains of chromosomes 7 and 17 combined with a loss of the Y chromosome. Additional numerical and structural chromosomal aberrations were observed in various combinations in all cell lines. None of our cell lines, however, exhibited the loss of a specific chromosomal region at chromosome 3p, known to be the characteristic chromosomal marker of the clear cell type of RCC (Yoshida *et al.*, 1986; Carrol *et al.*, 1987; Zbar *et al.*, 1987; Kovacs *et al.*, 1988). The gain of chromosomes 7 and 17 combined with the loss of the Y chromosome has been identified as the common denominator of the chromophilic (papillary) renal cell *carcinoma* and its supposed precursor lesion, the chromophilic (papillary) renal cell *adenoma* (Kovacs *et al.*, 1991; Kovacs, 1993). The malignant transformation of chromophilic renal cell tumours seems to be indicated by the acquisition of additional karyotype changes, such as polysomy of chromosomes 8, 12, 16 and 20 or loss of chromosomes 14, 21 and 22. It was interesting, therefore, to note that chromphi-2 cells exhibited only a minimum of additional chromosomal aberrations, including two marker chromosomes.

The specific combination of chromosomal aberrations in chromophilic RCCs, i.e. the loss of the Y chromosome and polysomy of chromosomes 7, 8, 12, 16, 17 and 20, suggests that genes located on these chromosomes are involved in the genesis of this specific type of renal cancer. According to the two-hit hypothesis (Knudson, 1987), the development of cancer might be associated with a sequence of genetic events resulting in the loss of both wild-type alleles of a tumour suppressor gene. Therefore, it has been hypothesised that a tumour-suppressor gene might be localised at the homologous regions of the X and Y chromosomes (Kovacs *et al.*, 1991; Kovacs, 1993). In this context, it is interesting to note the strong male preponderance of chromophilic (papillary) renal cell carcinoma, which has been estimated to be as high as 8 : 1 (Kovacs, 1993), and which also was evident in our investigation. As the mutational inactivation of the putative tumour-suppressor gene at the homologous regions of the X and Y chromosomes should occur at the same frequency by chance, other factors must contribute to the unequal sex distribution of chromophilic RCCs. A possible explanation might be derived from recent observations showing a high frequency of chromosomal mosaicism in normal kidney tissues obtained from patients with renal cancer. A clonal loss of the Y chromosome was observed in 21 out of 31 (68%) normal kidney probes obtained from men as opposed to clonal monosomy X in 1 out of 14 (7%) normal kidney probes obtained from women (Emanuel *et al.*, 1992). Therefore, precursor cells having only one allele of the putative suppressor gene seem to be more prevalent in male kidneys than in female kidneys, thus probably determining the male preponderance of chromophilic (papillary) RCC (Kovacs *et al.*, 1994).

On the other hand, the initiation of chromophilic tumour cells could also be related to the polysomy of chromosomes carrying mutant genes, as the amplification of mutant *vs*

**Figure 5** Representative karyotypes of chromphi-1 (**a**), chromphi-2 (**b**), chromphi-3 (**c**) and chromphi-4 (**d**).

**Table VII** Description of karyotypes in accordance with ISCN (1995)

| *Cell line* | *Description of karyotype (low passage number/high passage number)* |
|---|---|
| Chromphi-1 | Hypotriploid: 65~67, XX, −Y, del(1)(q25→qter)2x, der(1;?)(q10;?), +der(1;?)(q10;?),der(1;15)(q10;q10)2x, −1p, +2, der(2)del(2)(p21→pter)add(2)(q3?)2x, −3,add(3)(p25)2x, −4, add(6)(q2?), +7,der(?;8)(?;q10), −9, −11, +12,del(12)(q15?→qter)2x, −13,der(13)add(13)(q10)2x, −14,add(14)(q10)2x,add(15)(q10), +add(15)(q10), +16, −19, −20, −21, −22, +mar, +mar, +mar[cp20]/65~67, idem[cp10] |
| Chromphi-2 | Near diploid: 45~48,X, −Y, +7, +12, +der(17;21)(q10;q10), −21, +mar, +mar[cp20]/44~46,idem, −10, −mar, −mar[cp10] |
| Chromphi-3 | Near tetraploid:78~84,XX, −Y, −Y, −1, +5, −6,add(6)(q?), +7, +7, del(11)(q23→qter),del(11)(q22→qer), +14, +16, der(14?)t(14;17)(q12?;q21?)2x, +20, −21, −21, −21, −21, −22, −22, +mar, +mar, +mar,[cp20]/78~81,idem, −2, −add(6)(q?), −9, −9, +20, −22[cp10] |
| Chromphi-4 | Hypotriploid:62~66;XX, −Y,add(1)(p36), +2,del(4)(q3?→qter), +der(4;14)(q13;q22), +del(7)(q22→qter), −9, der(10)t(8;10)(q13?;p13?), +10,i(10)(q10), +17, −18, +20, +20, −21, +mar, +mar,[cp20]/60~64,idem, −i(10)(q10), −16, −19, +20[cp10] |

normal alleles has also been shown to affect cell proliferation and differentiation (Klein, 1981). So far, however, the genes actually affected by the polysomy of chromosomes 7, 8, 12, 16, 17 and 20 in chromophilic tumours remain to be defined.

In conclusion, the newly established cell lines represent a spectrum of chromophilic RCCs defined by distinct cytomorphological criteria and cytogenetic aberrations, not observed in the clear cell and chromophobe types of RCC. Therefore, these cell lines will become valuable tools for further investigations on the genetic, molecular and biological properties of chromophilic RCCs. Experimental studies analysing the invasive behaviour and the response to biological response modifiers in chromophilic RCCs are currently in progress in our laboratory.

**Acknowledgements**
We express our appreciation to Mrs A Florange-Heinrichs, Mrs H Balven, Mrs V Ludolf, Mrs M Müller, Mr P Pulkowsky, Mr M Ringler, Mr F Rinschede and Mrs H Auweiler for their excellent technical assistance. This work was supported by the Deutsche Forschungsgemeinschaft.

### References

ANGLARD P, TRAHAN E, LIU S, LATIF F, MERINO MJ, LERMAN MC, ZBAR B AND LINEHAN WM. (1992). Molecular and cellular characterization of human renal cell carcinoma cell lines. *Cancer Res.*, **52,** 348–356.

VAN DEN BERG E, VAN DER HOUT AH, OOSTERHUIS JW, STÖRKEL S, DUKHUIZEN T, DAM A, ZWEERS HMM, MENSINK HJA, BUYS CHCM AND DE JONG B. (1993). Cytogenetic analysis of epithelial renal cell tumors: relationship with a new histopathological classification. *Int. J. Cancer*, **55,** 223–227.

CARROL PR, MURTY VVS, REUTER V, JHANWAR S, FAIR WR, WHITMORE WF AND CHAGANTI RSK. (1987). Abnormalities at chromosome region 3p12–14 characterize clear cell renal carcinoma. *Cancer Genet. Cytogenet.*, **26,** 253–259.

EBERT T, BANDER NH, FINSTAD CL, RAMSAWAK RD AND OLD LJ. (1990). Establishment and characterization of human renal cancer and normal kidney cell lines. *Cancer Res.*, **50,** 5531–5536.

ENGERS R, GERHARZ CD, MOLL R, POHL A, SARBIA M AND GABBERT HE. (1994). Interclonal heterogeneity in a human epithelioid-sarcoma cell line (GRU-1). *Int. J. Cancer*, **59,** 548–553.

EMANUEL A, SZÜCS S, WEIER HUG AND KOVACS G. (1992). Clonal aberrations of chromosomes X,Y, 7 and 10 in normal kidney tissues of patients with renal cell tumors. *Genes Chrom. Cancer*, **4,** 75–77.

FINGERT HJ, CHEN Z, MIZRAHI N, GAJEWSKI WH, BAMBERG MP AND KRADIN RL. (1987). Rapid growth of human cancer cells in a mouse model with fibrin clot subrenal capsule assay. *Cancer Res.*, **47,** 3824–3829.

GERHARZ CD, GABBERT HE, ENGERS R, RAMP U, MAYER H, BIESALSKI HK AND LULEY C. (1989). Heterogenous response to differentiation induction in different clonal subpopulation of a rat rhabdomyosarcoma cell line (BA-HAN-1). *Cancer Res.*, **49,** 7132–7140.

GERHARZ CD, MOLL R, STÖRKEL S, RAMP U, THOENES W AND GABBERT HE. (1993). Ultrastructural appearance and cytoskeletal architecture of the clear, chromophilic and chromophobe types of human renal cell carcinoma in vitro. *Am. J. Pathol.*, **142,** 851–859.

GERHARZ CD, RAMP U, OLERT J, MOLL R, STÖRKEL S, MARX N AND GABBERT HE. (1994). Cytomorphological, cytogenetic and molecular biological characterization of four new human renal carcinoma cell lines of the clear cell type. *Virchows Arch.*, **424,** 403–409.

GERHARZ CD, MOLL R, STÖRKEL S, RAMP U, HILDEBARANDT B, MOLSBERGER G, KOLDOVSKY P AND GABBERT HE. (1995). Establishment and characterization of two divergent cell lines derived from a human chromophobe carcinoma. *Am. J. Pathol.*, **146,** 953–962.

GROSSMAN HB, WEDEMEYER G AND REN L. (1985). Human renal carcinoma: characterization of five new cell lines. *J. Surg. Oncol.*, **28,** 237–244.

HOEHN W AND SCHROEDER FH. (1978). Renal cell carcinoma: two new cell lines and a serially transplantable nude mouse tumour (NC 65). *Invest. Urol.*, **16,** 106–112.

HUGHSON MD, JOHNSON LD, SILVA FG AND KOVACS G. (1993). Non-papillary and papillary renal cell carcinoma: a cytogenetic and phenotypic study. *Modern Pathol.*, **6,** 449–456.

ISCN. (1995). *An International System for Human Cytogenetics Nomenclature*. Karger: Basle.

DEKERNION JB, RAMMING KP AND SMITH RB. (1978). The natural history of metastatic renal cell carcinoma: a computer analysis. *J. Urol.*, **120,** 148–152.

KLEIN G. (1981). The role of gene dosage and genetic transpositions in carcinogenesis. *Nature*, **294,** 313–318.

KNUDSON AG. (1987). A two mutation model of human cancer. *Adv. Viral Oncol.*, **7,** 1–17.

KOVACS G. (1989). Papillary renal cell carcinoma. A morphologic and cytogenetic study of 11 cases. *Am. J. Pathol.*, **134,** 27–34.

KOVACS G. (1993). Molecular cytogenetics of renal cell tumors. *Adv. Cancer Res.*, **63,** 89–124.

KOVACS G, ERLANDSON R, BOLDOG F, INGVARSSON S, MÜLLER-BRECHLIN R, KLEIN G AND SÜMEGI J. (1988). Consistent chromosome 3p deletion and loss of heterozygosity in renal cell carcinomas. *Proc. Natl Acad. Sci. USA*, **85,** 1571–1575.

KOVACS G, FUZESI L, EMANUEL A AND KUNG HF. (1991). Cytogenetics of papillary renal cell tumors. *Genes Chrom. Cancer*, **3,** 249–255.

KOVACS G, TORY K AND KOVACS A. (1994). Development of papillary renal cell tumours is associated with loss of Y-chromosomespecific DNA sequences. *J. Pathol.*, **173,** 39–44.

LATIF F, TORY K, GNARRA J, YAO M, DUH F, ORCUTT ML, STACKHOUSE T, KUZMIN I, MODI W, GEIL L, SCHMIDT L, ZHOU F, MING HL, WEI MH, CHEN F, GLENN G, CHOUKE P, WALTHER MM, WENG Y, DUAN DS, DEAN M, GLAVAC D, RICHARDS FM, CROSSEY PA, FERGUSON-SMITH MA, PASLIER D, CHUMAKOV I, COHEN D, CHINAULT AC, MAHER E, LINEHAN WM, ZBAR B AND LERMAN MI. (1993). Identification of the von Hippel–Lindau disease tumor suppressor gene. *Science*, **260,** 1317–1320.

MALDAZYS JD AND DEKERNION JB. (1986). Prognostic factors in metastatic renal carcinoma. *J. Urol.*, **136,** 376–379.

MATSUDA M, OSAFUNE M, NAKANO E, KOTAKE T, SONODA T, WATANABE S, HADA T, OKOCHI T, HIGASHINO K, YAMAMURA Y AND ABE T. (1979). Characterization of an established cell line from human renal carcinoma. *Cancer Res.*, **39,** 4694–4699.

MAYER D AND BANNASCH P. (1988). Activity of glycogen synthase and phosphorylase and glucose-6-phosphate content in renal clear cell carcinomas. *J. Cancer Res. Clin. Oncol.*, **114,** 369–372.

MOLL R, LOEWE A, LAUFER J AND FRANKE WW. (1992). Cytokeratin 20 in human carcinomas: a new histodiagnostic marker detected by monoclonal antibodies. *Am. J. Pathol.*, **140,** 427–447.

NAITO S, KANAMORI T, HISANO S, TANAKA K, MOMOSE S AND KAMATA N. (1982). Human renal cell carcinoma: establishment and characterization of two new cell lines. *J. Urol.*, **128,** 1117–1121.

NEVES R, ZINCKE H AND TAYLOR WF. (1988). Metastatic renal cell cancer and radical nephrectomy: identification of prognostic factors and patient survival. *J. Urol.*, **139,** 1173–1176.

PITZ S, MOLL R, STÖRKEL S AND THOENES W. (1987). Expression of intermediate filament proteins in subtypes of renal cell carcinomas and in renal oncocytomas. *Lab. Invest.*, **6,** 642–653.

SEABRIGHT M. (1971). A rapid banding technique for human chromosones. *Lancet*, **2,** 971–972.

STEINBERG P, STÖRKEL S, OESCH F AND THOENES W. (1992). Carbohydrate metabolism in human renal clear cell carcinomas. *Lab. Invest.*, **67,** 506–511.

STÖRKEL S, STEART PV, DRENCKHAHN D AND THOENES W. (1989). The human chromophobe cell renal carcinoma: its probable relation to intercalated cells of the collecting duct. *Virchows Arch. B Cell Pathol.*, **56,** 237–245.

SYTKOWSKI AJ, RICHIE JP AND BICKNELL KA. (1983). New human renal carcinoma cell line estblished from a patient with erythrocytosis. *Cancer Res.*, **43,** 1415–1419.

THOENES W, STÖRKEL S AND RUMPELT HJ. (1986). Histopathology and classification of renal cell tumors (adenomas, oncocytomas and carcinomas). *Path. Res. Pract.*, **181,** 125–143.

THOENES W, STÖRKEL S, RUMPELT HJ, MOLL R, BAUM HP AND WERNER S. (1988). Chromophobe cell renal carcinoma and its variants - a report on 32 cases. *J. Pathol.*, **155,** 277–287.

VINDELOV L, CHRISTENSEN IJ AND NISSEN NI. (1983). Standardization of high-resolution flow cytometric DNA analysis by the simultaneous use of chicken and trout red blood cells as internal reference standards. *Cytometry*, **5,** 328–331.

WORLD HEALTH ORGANIZATION. (1981). Histological typing of kidney tumours. In *International Histologic Classification of Tumours*, no. 25, Mostofi FK. (ed.) pp. 15–26 World Health Organization: Geneva.

YOSHIDA MA, OTHYASHIKI K, OCHI H, GIBAS Z, PONTES JE, PROUT GR AND SANDBERG AA. (1986). Cytogenetic studies of tumor tissue from patients with nonfamilial renal cell carcinoma. *Cancer Res.*, **46,** 2139–2147.

ZBAR B, BRAUCH H, TALMADGE C AND LINEHAN M. (1987). Loss of alleles of loci on the short arm of chromosome 3 in renal cell carcinoma. *Nature*, **327,** 721–724.

ZBAR B, TORY K, MERINO M, SCHMIDT L, GLENN G, CHOYKE P, WALTHER MM, LERMAN M AND LINEHAN WM. (1994). Hereditary papillary renal cell carcinoma. *J. Urol.*, **151,** 561–566.